# Occurence of Post-Traumatic Stress Symptoms, Anxiety and Depression in the Acute Phase of Transient Ischemic Attack and Stroke

**DOI:** 10.1007/s11126-020-09873-9

**Published:** 2021-01-02

**Authors:** Helge H. O. Müller, Katharina Czwalinna, Ruihao Wang, Caroline Lücke, Alexandra P. Lam, Alexandra Philipsen, Jürgen M. Gschossmann, Sebastian Moeller

**Affiliations:** 1 Universitätsklinikum Bonn AöR, Klinik und Poliklinik für Psychiatrie, Bonn, Germany; 2 Faculty of Health/School of Medicine, Integrative Psychiatry and Psychotherapy, Witten/Herdecke University, Witten, Germany; 3 Abteilung für Psychiatrie und Psychotherapie, Gemeinschaftskrankenhaus Herdecke gGmbH, Herdecke, Germany; 4 Klinikum Forchheim - Fränkische Schweiz gGmbH, Forchheim, Germany; 5 Friedrich-Alexander Universität Erlangen-Nürnberg, Erlangen, Germany; 6 Klinik und Poliklinik für Neurologie, Universitätsklinikum Erlangen, Erlangen, Germany

**Keywords:** PTSD, TIA, Stroke, Myocardial infarction, Depression, Anxiety, Coping strategies, HRQoL

## Abstract

Rates of post-traumatic stress symptoms, anxiety and depression are increased in patients having experienced a transient ischemic attack (TIA) or stroke several months ago. However, data of psychiatric symptoms in the acute phase within the first days after ictus are lacking. In 20 patients with stroke and 33 patients with TIA we assessed disease severity by means of the NIHSS, levels of depression and anxiety by HADS, PTSD-like symptoms by PC-PTSD, quality of life (HrQoL) by SF-12, and coping style by brief COPE Inventory within the first 5 days after ictus. NIHSS on admission was lower in patients with TIA (0 ± 1) than in patients with stroke (3 ± 2, *p* < 0.001). HADS depression score was significantly higher in patients with stroke (7.0 ± 4.5) than in patients with TIA (4.9 ± 4.0). HADS anxiety score, HrQoL and coping styles were similar between TIA and stroke patients (*p* > 0.05). 5 and 3 of 33 TIA patients as well as 4 and 3 of 20 stroke patients had at least 11 points in the HADS anxiety and depression score respectively (*p* = 0.001). 2 of 33 TIA patients and 2 of 20 stroke patients had more than 2 points in the PC-PTSD (*p* = 0.646). We did not find consistent correlations between the NIHSS and the psychometric parameters. Within the first five days after patients having experienced a TIA or stroke PTSD-like, anxious and depressive symptoms are more common than in the general population. As the acute psychological status after ictus is predictive for psychiatric comorbidity years later physicians should pay attention and adequately treat psychiatric symptoms already in the acute phase of stroke.

**Trial Registration**: German Clinical Trials Register, DRKS00021730, https://www.drks.de/drks_web/navigate.do?navigationId=trial.HTML&TRIAL_ID=DRKS00021730, registered 05/19/2020- Retrospectively registered.

## Background

Transient Ischemic Attack (TIA) and stroke are common neurological conditions. In the US, TIAs are diagnosed annually between 200,000 and 500,000 [1]. According to a report from the Global Burden of Disease, the estimated global lifetime risk of stroke for those aged 25 years or older was 24.9% [2]. TIA is defined as transient episode of neurologic dysfunction caused due to loss of blood flow to the brain or spinal cord without acute infarction. In contrast to TIA which is usually short-lived, in stroke there is a permanent tissue damage [3]. Depending on the area of the brain involved, TIA and stroke symptoms may include numbness, weakness or paralysis, slurred speech, aphasia or blurred vision [4, 5]. TIA is not associated with permanent neurological deficits. In contrast, stroke survivors may have permanent deficits, e.g., aphasia, weakness or hemiparesis. TIA and stroke are described by a dramatic and sudden onset [3, 5]. Both conditions may pose the risk of experiencing a sudden traumatic occurrence of neurological symptoms during ictus. This experience is supposed to contribute to higher rates of posttraumatic stress disorder (PTSD) related symptoms and also higher rates of subsequent depression and anxiety [4].

The Diagnostic and Statistical Manual of Mental Disorders in the fifth edition (DSM-V) defines PTSD as exposing to actual or threatened serious injury, intrusive symptoms associated with the traumatic event, frequent avoidance of reminders associated with the traumatic event, changes in thoughts and mood that occurred or worsened following the traumatic event, changes in arousal that started or worsened following the event, the symptoms bring distress and/or interfere with a number of different areas of life [6]. PTSD related symptoms may occur in patients after ictus [7].

Additionally, in stroke patients the residual impairment and sequelae of permanent neurological deficits have impact on quality of life, and contributes to psychological and psychosocial problems [8, 9]. Thus, increased prevalence rates of depression and anxiety are found in stroke survivors [4, 10–12].

The prevalence rate of chronic PTSD after stroke and TIA can be estimated between 11 and 28%, depending on the time-point and method of assessment [4, 13–15]. Moreover, patients with chronic PTSD tend to have higher rates of depression, anxiety, maladaptive coping and reduced health related quality of life compared to patients without PTSD [4, 10–12].

In general, only a few studies assessed the prevalence of PTSD-like syptoms, anxiety and depression due to the occurrence of stroke or TIA several months to years after ictus [4, 10–12]. Moreover, data of coping strategies in these patients are even rarer, which is suprising knowing the cross link of depressive and PTSD-like symptoms to hampered outcomes [12, 16, 17]. However, data of the aferomentioned psychiatric symptomps in the acute phase within the first days after ictus are lacking. Therefore, the current prospective study sought to explore levels of depression and anxiety, PTSD-like symptoms, quality of life, and coping style with standardized questionnaires within the first 5 days after TIA or stroke. Additionally, the neurological status of the patients were documented.

## Methods

### Study Sample

The study was conducted in colloboration at the Stroke Unit / Intermediate Care Unit of Klinikum Forchheim - Fränkische Schweiz gGmbH between 2017 and 2018.

The Klinkum Forchheim is a medium-sized general hospital in Franconia, Germany. The study was approved by the Ethics Committee of the University of Oldenburg (Registration-Nr.: 2016–169) and performed in accordance with the ethical standards as set forth in the Declaration of Helsinki and its later amendments. The study is registered in the German Clinical Trials Register (DRKS00021730). We examined 66 consecutive patients hospitalised at stroke unit / intermediate care unit due acute ischaemic or hemorrhagic stroke. All subjects gave their written informed consent prior to their voluntary participation in the study. In total, 53 patients were included in the study after we excluded patients due to severe cognitive impairment (*n* = 7), missing capacity to provide informed consent due to aphasia (*n* = 5) or previous PTSD (*n* = 1).

### Psychometric Assessment

The following questionnaires and scoring systems were used:

#### Primary Care-PTSD Screen (PC-PTSD)

The German version of the Primary Care-PTSD Screen (PC-PTSD) was used to assess the occurrence of PTSD symptoms. The PC-PTSD is a validated brief 4-item instrument developed by the Veteran’s Administration to screen for PTSD in primary care settings [18]. It asks: “In your life, have you ever had any experience that was so frightening, horrible, or upsetting that, in the past month you…” This statement is followed by four items that broadly correspond to above mentioned DSM-V diagnostic criteria: intrusion; avoidance, hyperarousal and numbness or detachment (from others, activities, or surroundings). A binary cut-off score of >2 was used in our study [18].

#### Hospital Anxiety and Depression Scale (HADS)

The degree of depression and anxiety during the preceding week was assessed using the German version of the hospital anxiety and depression scale (HADS) [19]. Separate anxiety and depression subscale scores can be assessed. HADS scores were categorised as normal (0–7), borderline abnormal (8–10) and abnormal (11–21) [19].

#### Short Form (SF)-12

Quality of life (HRQoL) was assessed using the german version of the Short Form (SF)-12 questionaiere. The SF-12 uses only 12 of the 36 questions from the SF-36, and the 12 questions can be abstracted from the answers provided in the SF-36 [20]. The 12 items are aggregated into two health summary scales that reflect physical (PCS) and mental (MCS) components ranging from 0 (worst) to 100 (best). The component scores for the SF-12 are norm-based, with a mean score of 50 and standard deviation of ±10. A higher score reflects better HRQoL and a lower score, poorer HRQoL [20].

#### Brief COPE Inventory (COPE)

The German version of the brief COPE Inventory was applied to assess patient’s disposition for using maladaptive coping strategies [21]. The sum of maladaptive coping strategy scores (denial, substance use, behavioural disengagement, self-distraction, self-blame) was assessed as an indicator of maladaptive coping. The sum of the adaptive coping strategy scores (active coping, emotional support, expression of emotions, instrumental support, positive reinterpretation, planning, humor and acceptance) was assessed as an indicator of adaptive coping. The higher the maladaptive or adaptive coping test score, the more patients habitually use maladaptive or adaptive coping strategies, respectively [22].

#### Somatic Diagnostics

We assessed clinical stroke severity by means of the NIHSS (ranging from 0 to 42 points) [23] on admission and on discharge. Moreover, all patients received a questionnaire to assess sociodemographic data and information about their medical history, i.e. previous stroke/TIA, rheumatic disease, diabetes mellitus (DM), coronary artery disease, dementia, thyroid disease or cancer.

#### Statistics

For data analysis, we used a commercially available statistical program (SPSS™ 21.0, SPSS Inc., Chicago, IL). Data were tested for normal distribution using the Shapiro-Wilk test. For comparison of the NIHSS, PTSD-like symptoms, HADS depression and anxiety score, SF-12 and COPE between patients with TIA and patients with stroke, we used the Mann–Whitney U-test. For dichotomous parameters, such as presence or absence of previous stroke/TIA, rheumatic disease, diabetes, coronary artery disease, dementia, thyroid disease or cancer, psychopharmacotherapy on admission and discharge, PTSD-like symptoms, normal, borderline abnormal and abnormal test results in the HADS in patients with TIA and patients with stroke, we used Chi-squared test or Fisher exact test as appropriate. Correlation between NIHSS scores and the different psychometric assessment results was assessed with the Spearman rank correlation test. We used an adjusted binary logistic regression analysis to calculate the odds ratios and 95% confidence intervals of PTSD-like symptoms with previous psychiatric disorders and sociodemographic data as covariates.

## Results

We examined 20 patients with stroke and 33 patients with TIA. Demographical patient data are summarized in Table 1. NIHSS on admission was lower in patients with TIA (0 ± 1) than in patients with stroke (3 ± 2, *p* < 0.001). HADS depression score was significantly higher in patients with stroke (7.0 ± 4.5) than in patients with TIA (4.9 ± 4.0, *p* = 0.048). HADS anxiety score, PCS, MCS and coping styles were similar between TIA and stroke patients (*p* > 0.05, Table 2). 10 of 33 TIA patients and 7 of 20 stroke patients have had a stroke or TIA before (*p* < 0.001). For other comorbid diseases in the TIA and stroke patients, i.e., rheumatic disease, DM, coronary artery disease, dementia, thyroid disease or cancer, see Table 3. 4 of 32 TIA patients and 2 of 20 stroke patients had psychopharmacotherapy on admission (*p* < 0.001). The same 4 of 33 TIA patients and the same 2 of 20 stroke patients had psychopharmacotherapy on discharge (p < 0.001). 2 of 33 TIA patients and 2 of 20 stroke patients had more than 2 points in the PC-PTSD (*p* = 0.646). 21 and 24 of 33 TIA patients as well as 12 and again 12 of 20 stroke patients had between 0 and 7 points in the HADS anxiety and depression score respectively (*p* < 0.001). 5 and 4 of 33 TIA patients as well as 4 and 5 of 20 stroke patients had between 8 and 10 points in the HADS anxiety and depression score respectively (p < 0.001). 5 and 3 of 33 TIA patients as well as 4 and 3 of 20 stroke patients had at least 11 points in the HADS anxiety and depression score respectively (*p* = 0.001).

**Table 1:** Descriptive sample characteristics

	Total sample	TIA patients (*n* = 32)	Stroke patients (*n* = 20)	*p* value
Age (years)	70.0 ± 11.8	68.6 ± 13.4	72.2 ± 8.1	0.09
Sex (fem./male) n (%)	25 (48%)/ 27 (52%)	17 (53%)/ 15 (47%)	8 (40%)/ 12 (60%)	0.40
Average number of children	1.5 ± 1.3	1.7 ± 1.1	1.3 ± 1.5	0.47
Without partnership/ Widow(er) n (%)	10 (19%)	4 (13%)	6 (30%)	0.07
With partership/married	42 (81%)	28 (88%)	14 (70%)	
Retired/not retired n (%)	36 (69%)/ 16 (31%)	20 (63%)/ 12 (38%)	16 (80%)/ 4 (20%)	0.23

*fem* female

**Table 2:** HADS depression and anxiety score, physical and mental score of the SF-12, active-functional, cognitive-functional and dysfunctional coping scores of the COPE in comparisson between TIA and stroke patients

	Total sample (*n* = 52)	TIA patients (n = 32)	Stroke patients (n = 20)	p value
HADS-D	5.7 ± 4.3	4.9 ± 4.0	7.0 ± 4.5	**0.048**
HADS-A	6.0 ± 4.1	6.0 ± 4.0	6.0 ± 4.2	0.932
PCS	36.4 ± 11.6	36.0 ± 11.8	36.0 ± 12.3	0.625
MCS	50.1 ± 10.1	49.4 ± 11.2	51.0 ± 10.8	0.560
coping				
active-f.	17.9 ± 7.9	17.7 ± 7.9	18.3 ± 6.8	0.962
cognitive-f.	14.7 ± 6.0	14.5 ± 6.0	14.9 ± 4.4	0.902
dysfunctional	8.8 ± 3.8	8.7 ± 3.8	9.5 ± 3.3	0.550

*HADS-D* Hospital Anxiety and Depression Scale Depression score, *HADS-A* Hospital Anxiety and Depression Scale Anxiety score, *PCS* physical component score of the Short Form (SF)-12, *MCS* mental component score of the Short Form (SF)-12, *f* functional

**Table 3:** Number of TIA and stroke patients (%) with TIA or stroke in the medical history. Comormid diseases, i.e., rheumatic disease, diabetes mellitus, coronary artery disease, dementia, thyroid disease and cancer

Previous TIA/stroke Comorbid disease	Total sample (n = 52)	TIA patients (n = 32)	Stroke patients (n = 20)	p value
TIA/stroke	17 (32%)	10 (31%)	7 (35%)	**<0.001**
rheumatic disease	4 (8%)	3 (9%)	1 (5%)	0.472
DM	20 (39%)	10 (31%)	10 (50%)	0.324
CAD	28 (54%)	16 (50%)	12 (60%)	0.611
dementia	3 (6%)	2 (6%)	1 (5%)	0.710
Thyroid disease	12 (23%)	9 (28%)	3 (15%)	0.372
cancer	10 (19%)	6 (19%)	4 (20%)	0.726

*DM* diabetis mellitus, *CAD* coronary artery disease

HADS depression score only inversely correlated with NIHSS on admission (Rho = −0.281, *p* < 0.048) but not with NIHSS on discharge (*p* > 0.05). PCS only inversely correlated with NIHSS on admission (Rho = −0.285, *p* < 0.041) but not with NIHSS on discharge (p > 0.05). Cognitive coping style correlated with NIHSS on admission (Rho = 0.804, p < 0.001) and NIHSS on discharge (Rho = 0.597, p < 0.001). HADS anxiety score, MCS, active coping style and dysfunctional coping style did neither correlate with NIHSS on admission nor NIHSS on discharge. In the binary logistic regression analysis model only previous psychiatric disorder had an odds on the prevalence of PTSD-like symptoms (Odds ratio 15.0, 95% Confidence Interval 1.531–146.937, *p* = 0.002, Table 4).

**Table 4:** Binary logistic regression analysis between posttraumatic stress disorders and covariates among 53 patients after stroke or TIA

Covariate	Odds ratio	95% Confidence Intervals	p Value
Age	0.979	0.914–1.049	0.552
Gender, men vs. women	4.174	0.434–40.188	0.216
Children, with vs. without	0.514	0.076–3.458	0.494
Education, with vs. without	0.671	0.163–2.757	0.580
Career, with vs. without	0.372	0.033–4.180	0.423
Current working status, active vs. non-active	1.744	0.261–11.657	0.566
Previous psychiatric disorders, with vs. without	15.000	1.531–146.937	**0.020**

## Discussion

We have shown, that even within the first five days after patients having experienced a TIA or stroke PTSD-like, anxious and depressive symptoms may occur. Previous research had focused on PTSD, anxiety and depression month to years after ictus [4, 10–12]. In a TIA-only sample Kiphuth et al. found a PTSD prevalence of 29% in patients having experienced a TIA at least 3 months ago [4]. Stein et al. reported a PTSD prevalence of 11% 6–12 months after first ischemic stroke [13]. Kronish et al. [14] and Goldfinger et al. [15] stated a PTSD prevalence of 18% in patients having experienced a stroke or TIA in the previous 5 years. In our total patient cohort of acute TIA and Stroke patients the prevalence of PTSD-like symptoms was 8% with similar rates in both patient groups. Thus, the prevalence rate of PTSD-like symptoms is higher than the lifetime prevalence in the general population in Europe (0.56% to 6.67%) [24] but lower than the prevalence rates in patients who have had the TIA or stroke several months or years ago [4, 13–15, 25]. Neurobiological mechanisms, i.e., alterations in the noradrenergic, serotonergic, endogenous cannabinoid and opioid systems as well as the hypothalamic-pituitary adrenal axis, with maladaptive psychobiological responses over time to the trauma of the ictus may explain our lower prevalence rate compared with studies who examined the patients at least several months after the trauma [26]. Previous psychiatric diagnosis but not demographical patient characteristics, e.g. age, sex or educational level, had an odds on the prevalence of PTSD-like symptoms in our patient cohort. This is in line with results from the literature, according to which, e.g., approximately 50% of the patients with PTSD also suffer from depression [27]. 15 of our 53 patients with TIA and Stroke had at least 8 points in the HADS depression score on discharge suggesting that almost 30% of the patients are at least suggestive of the presence of depressive symptoms [28]. Post-stroke depression (PSD) can be ascribed as the most prevalent psychiatric complication of acute stroke [29, 30]. PSD affects about 30% of patients within a five-year period after stroke [29, 31, 32]. Schöttke et al. determined a PSD prevalence of 32.2% several weeks after ictus [25]. Our findings of similar depression rates in TIA and stroke patients confirm the suggestion of Douven et al. that the generalized degenerative and vascular brain pathology, rather than lesion-related pathology, is a predictor for the development of PSD [33]. Higher mean values of HADS depression score in our stroke patients than in our TIA patients is not a contradiction to the conclusion of Douven et al. [33] because our sample of stroke patients was numerically older than the TIA patients although age was not quite significantly higher in stroke patients than in TIA patients (*p* = 0.09). Subsequently degenerative brain processes may be more common in our stroke patients than in our TIA patients [34]. The fact that only 6 of our patients (11%) received psychopharmacotherapy on discharge, even though 15 patients (28%) showed depressive symptoms, emphasizes that PSD is still an underdiagnosed disease in everyday clinical practice that is not given enough attention [30]. Schöttke et al. showed that the PSD status in the subacute phase of stroke significantly predicted the PSD status 3 years later with a fivefold higher long-term PSD-risk [25]. In contrast to anxiety disorder, depression increases the mortality and disability of stroke patients [35]. Thus, detection of high-risk patients in the acute stroke phase who may develop PSD-symptoms in the further course would be desirable. Only early detection of depressive symptoms may enable fast and effective therapy. This is the only way to prevent further chronification, delay of therapy and prolongation of the length of hospital stay subsequently followed by a poorer prognosis [32]. The finding of similar PCS, MCS and coping styles between our TIA and stroke patients support the conclusion of previous studies that there is not consistently a direct correlation between functional disability and the subjective HRQoL [36]. Actually, psychological factors may modify the perception of individual well-being regardless of disability degree. Previous research proposed that there is positive impact of active- and task-oriented coping strategies on HRQoL [36].

We have not found consistent correlations between the NIHSS, i.e., a marker of severity of disability in patients with acute stroke [23], and the psychometric findings, e.g., HADS, MCS, PCS, active coping style and dysfunctional coping style. Only cognitive coping style correlated with NIHSS on admission and on discharge. Herrmann et al. concluded from their comparison between patients with malignant brain tumors, stroke, Parkinson’s disease and traumatic brain injury that coping styles are mainly related to age and social factors and less related to the type of brain pathology [37]. Again, our findings of no consistent correlations could be related to the fact that there is generally no congruent interrelationship between functional disability and various psychometric parameters [37]. However, the inconsistent correlations could also be related to our small sample size.

Therefore, the findings of our study need to be interpreted cautiously due to the heterogeneity of the patient groups and the overall small sample size. The limitations of our study should be resolved through further assessments with larger and more homogenous patient cohorts. Another limitation of our study is the definition of PTSD-like symptoms. Actually, full diagnostic criteria of PTSD are not met until at least six months after the trauma, although onset of symptoms may occur immediately [6]. Thus, it is unclear whether our patients with PTSD-like symptoms will develop the full clinical picture of PTSD in the future. Nevertheless, this is precisely why there is urgent need to closely follow-up this potential risk group.

To conclude, within the first five days after patients having experienced a TIA or stroke PTSD-like, anxious and depressive symptoms are more common than in the general population [24]. Our observed findings let to the conclusion that there are no consistent correlations between the severity of disability in patients with acute stroke and psychiatric and psychological symptoms. This finding again emphasizes the need for further psychiatric observation, especially of this group.

## Conclusion

After TIA or stroke PTSD-like, anxious and depressive symptoms occur and are more common than in the general population. As the acute psychological status after ictus is predictive for psychiatric comorbidity years later physicians should pay attention and adequately treat psychiatric symptoms already in the acute phase of TIA and stroke.

## Data Availability

The datasets generated during and analyzed during the current study are available from the corresponding author on reasonable request.

## Abbreviations

TIA: Transient ischemic attack
NIHSS: National Institutes of Health Stroke Scale
HADS: Hospital Anxiety and Depression Scale
PTSD: Post-traumatic stress disorder
HRQoL: Health-related quality of life
SF-12: Short Form 12 Health Survey
COPE: Coping Strategies Inventory
DSM-V: Diagnostic and Statistical Manual of Mental Disorders fifth edition
PC-PTSD: Primary Care Checklist for post-traumatic stress disorder
DM: Diabetes mellitus
PSD: Post-stroke depression
PCS: Physical component summary
MCS: Mental component summary
SPSS: Statistical Package for the Social Sciences
e.g.: exempli gratia, for example
i.e.: id est., that is
